# A Case of Polymicrobial, Gram-Negative Pulmonic Valve Endocarditis

**DOI:** 10.1155/2019/6439390

**Published:** 2019-03-28

**Authors:** Tatiana Bekker, Anusha Govind, Devin M. Weber

**Affiliations:** ^1^Department of Medicine, Sidney Kimmel Medical College, Thomas Jefferson University, Philadelphia, PA, USA; ^2^Department of Medicine, Division of Infectious Diseases, Sidney Kimmel Medical College, Thomas Jefferson University, Philadelphia, PA, USA

## Abstract

Infective endocarditis due to Gram-negative, non-HACEK bacteria is a rare clinical entity. Even moreso, isolated pulmonic valve endocarditis accounts for less than 1.5–2% of all cases of infective endocarditis. These disease pathologies commonly occur in the setting of intravenous drug abuse, indwelling catheters or cardiac devices, or underlying structural heart disease. We present a unique case of pulmonic valve endocarditis in the setting of persistent polymicrobial bacteremia with *Klebsiella pneumoniae* and *Citrobacter koseri* with recent gastrointestinal instrumentation evolving into isolated pulmonic valve endocarditis.

## 1. Introduction

Infective endocarditis (IE) is an infrequent disease with a reported annual incidence of 3–7 cases per 100,000 person years [1]. Gram-positive organisms, such as staphylococci and streptococci, are identified in the overwhelming majority of all cases. Gram-negative organisms are uncommon causes of endocarditis as they are typically less adherent to heart valves and highly sensitive to bactericidal antibiotics [2, 3]. Herein, we present a case of isolated pulmonic valve endocarditis with the polymicrobial pathogens *Klebsiella* and *Citrobacter* spp.

## 2. Case Presentation

A 57-year-old male with a history of sickle cell disease (HbSC) without long-term indwelling vascular access complicated by recurrent cholecystitis and choledocholithiasis presented to our quaternary care hospital with a chief complaint of right upper quadrant and back pain. His vital signs on presentation included a temperature of 99.2°F, heart rate of 107 beats per minute, blood pressure of 105/58, respiratory rate of 22/min, and oxygen saturation of 96% on room air. His exam was remarkable for scleral icterus and right upper quadrant tenderness. Initial labs were notable for a leukocytosis of 38,200 cells/mm^3^, direct hyperbilirubinemia of 26.1 mg/dL, aspartate aminotransferase (AST) and alanine aminotransferase (ALT) of 425 IU/L and 303 IU/L, respectively, and a lactate of 7.3 mmol/L. He was admitted to the intensive care unit for management of shock. Blood cultures drawn on the day of admission grew *Klebsiella pneumoniae* and *Citrobacter koseri*. He was started on intravenous piperacillin-tazobactam. Imaging at the time of presentation displayed evidence of prior cholecystectomy with obstructive choledocholithiasis and multiple liver abscesses less than 2 cm in diameter. He underwent emergent endoscopic retrograde cholangiopancreatography (ERCP) with balloon dilation of the common bile duct and biliary stent placement. Transthoracic echocardiogram done at that time showed decreased ejection fracture with otherwise normal valve structure and function. Despite biliary stenting, the patient continued to complain of right upper quadrant and lower back pain and his blood cultures remained persistently positive after stenting.

**Figure 1:**
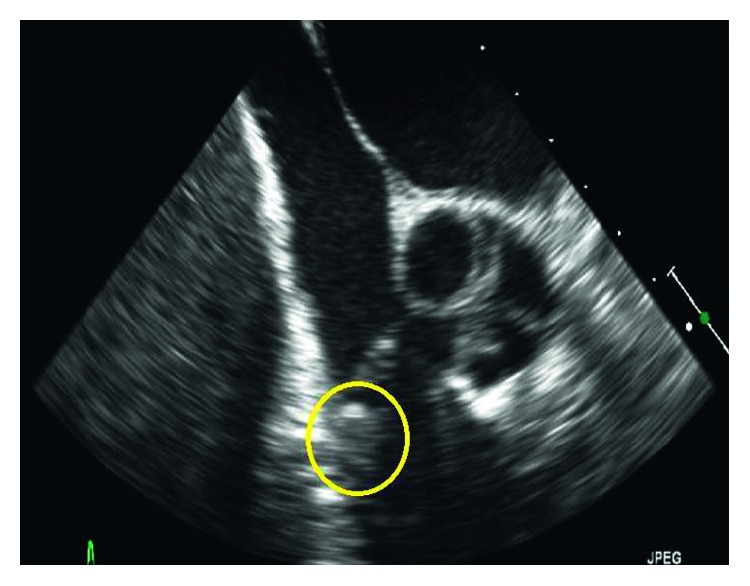
Transesophageal echocardiogram displaying a 10 mm mobile mass appreciated on 2D and 3D views consistent with vegetation (yellow circle).

On hospital day 6, the patient was reimaged with magnetic resonance cholangiopancreatography (MRCP) which displayed evidence of possible ongoing biliary obstruction. A midline was placed for ease of access for medication administration. On hospital day 7, repeat ERCP showed a patent stent from his prior procedure, but a second stent was placed to relieve a left hepatic lobe stricture. Even with this attempted source control, polymicrobial bacteremia persisted with both *Klebsiella pneumoniae* and *Citrobacter koseri* through hospital day 12. A transesophageal echocardiogram (TEE) was completed to evaluate for infectious endocarditis as an ongoing source of bacteremia. A 10 mm mobile vegetation was visualized on the pulmonic valve with mild pulmonary regurgitation (Figure 1). The Infectious Diseases consult team advised switching from piperacillin-tazobactam to combination therapy of intravenous ceftriaxone and oral ciprofloxacin. His midline catheter was removed, but tip was not sent for culture. His blood cultures cleared on hospital day 13. Repeat CT abdomen pelvis completed on hospital day 18 revealed an overall improvement of the previous liver abscess, except for one right lobe abscess that was slightly increased in size, and a new subpleural left lower lung lobe nodule concerning for septic embolus. Surgical evaluation was not pursued given his comorbidities and poor surgical candidacy. Ultimately, he was discharged to complete 6 weeks of intravenous ceftriaxone and oral ciprofloxacin. At the time of this case report, he has not required further intervention on the heart valve.

## 3. Discussion

Gram-negative bacteremia is typically transient, especially once appropriate antibiotic therapy is initiated. In addition, although transient bacteremia has been implicated in the setting of gastrointestinal instrumentation, seldom has this been linked to persistent bacteremia and even less have cases been associated with a complication of endocarditis. Thus, routine antibiotic prophylaxis is not indicated for such procedures, particularly in patients without any underlying valvular disease such as our patient [4]. Persistent Gram-negative bacteremia (>7 days) is infrequently encountered and should raise suspicion for endocarditis [5]. Polymicrobial right-sided endocarditis is uncommon but has been described most frequently in the intravenous drug abuser population. Isolated pulmonic-valve infective endocarditis is a rare entity, comprising less than 1.5–2% of all infective endocarditis cases [2, 6].

The International Collaboration on Endocarditis evaluated 2761 cases of infectious endocarditis as a prospective cohort study from 2000 to 2005. Gram-negative endocarditis accounted for about 1 to 10% of cases, with HACEK organisms occupying most of those. Non-HACEK Gram-negative organisms were identified in less than 1.8% of all endocarditis cases, with the most common organisms being *Escherichia coli* and *Pseudomonas aeruginosa*. 57% of these cases were healthcare associated and often related to implanted endovascular devices or intravenous drug abuse. For all Gram-negative organisms, treatment with a 6-week course of combination antibiotic therapy, such as a beta-lactam plus an aminoglycoside or fluoroquinolone, along with cardiac surgery evaluation is recommended [1, 6, 7].

*Klebsiella pneumoniae* rarely causes endocarditis and accounts for only 10% of non-HACEK Gram-negative endocarditis cases. In retrospective case reviews from the Minneapolis Veterans Administration system between 1993 and 1997, *Klebsiella* spp. were found to be the third leading cause of bacteremia, with endocarditis complicating only 1.2% of cases. None of those cases identified pulmonic valve disease. A more comprehensive literature review by the same authors, confirmed that not only is *Klebsiella* endocarditis rare but carries an ominous prognosis, with reported overall mortality rates as high as 49%. In that review, patients were managed both with prolonged course of antibiotics alone or with surgical intervention in addition to medical therapy. Improved outcomes and mortality benefit were noted in patients who underwent surgical interventions [8].

*Citrobacter* spp. have likewise been reported in a limited number of cases of endocarditis. Of these, disease was more commonly right-sided. Many, but not all, had predisposing risk factors for Gram-negative endocarditis such as indwelling catheter and intravenous drug abuse. The majority of cases appear to have been managed with a prolonged course of parenteral antibiotics with good outcomes [9, 10].

Finally, it is worth discussing that our patient's history of HbSC posed an additional management challenge and distinctive qualifier in our case. In general, for non-HACEK Gram-negative endocarditis, combined cardiac surgery with a prolonged course of antibiotics is recommended [1]. In patients with HbSC, however, cardiac surgery is associated with a particularly high morbidity and mortality largely related to an increased risk of precipitating crises [11].

Gram-negative, isolated pulmonic valve endocarditis in the setting of polymicrobial bacteremia after gastrointestinal procedural instrumentation is exceptionally uncommon. Treatment often involves both medical and surgical interventions. Our case is unique as it highlights the development of this infection in a patient with ongoing gastrointestinal issues and underscores the management challenges of underlying comorbidities.
